# PD40 Pilot Healthcare Programs - Bridging The Evidence Gap For Innovative Technologies

**DOI:** 10.1017/S0266462324003039

**Published:** 2025-01-07

**Authors:** Jarosław Gruszka, Katarzyna Sejbuk-Rozbicka, Magdalena Koperny, Maciej Dzik, Aleksandra Pelczarska, Joanna Syta, Kamila Malinowska

## Abstract

**Introduction:**

The scarcity of high quality evidence is a common constraint on the willingness to publicly fund innovative technologies. Our aim was to prepare a Methods and Process Guide to support the development of pilot healthcare programs in Poland. Such guides play a pivotal role in enhancing the quality of pilot programs and confidence in public funding decisions in health care.

**Methods:**

We reviewed guidelines for pilot healthcare programs published by the World Health Organization (WHO) and other healthcare organizations and analyzed the pilot healthcare programs in Poland. The Ministry of Health in Poland and the general public will be invited to provide feedback on the Guide.

**Results:**

Pilot programs serve as valuable testing grounds for healthcare solutions in low risk, small-scale clinical practice settings. A pilot program may be considered for interventions with proven safety and effectiveness, and when the intervention is complex, its implementation requires testing, or the intervention is considered high cost. Our Methods and Process Guide defines key elements of pilot healthcare programs, including objectives, starting criteria, conducting conditions, and monitoring rules. Public consultation on the Guide is underway.

**Conclusions:**

The publicly available Methods and Process Guide should enhance the methodological rigor of pilot healthcare programs in Poland. Well-designed pilot programs are expected to provide high quality real-world data that will facilitate public funding decisions for innovative technologies.

